# State of the Art in the Treatment of Congenital Agenesis With Implant-Supported Prosthesis: A Comprehensive Multidisciplinary Management

**DOI:** 10.1155/2024/5901688

**Published:** 2024-10-17

**Authors:** Gabriela Ezequiel Oliveira, Henrique Cassebe Ledo Pelegrine, Luiz Antonio B. Barros-Filho, Mauricio de Almeida Cardoso, Weber Adad Ricci, Luiz Antonio Borelli Barros, Erica Dorigatti de Avila, Rafael Scaf de Molon

**Affiliations:** ^1^Department of Diagnosis and Surgery, School of Dentistry, São Paulo State University (UNESP), Rua Jose Bonifacio, 1193. Vila Mendonça, Aracatuba, São Paulo 16015-050, Brazil; ^2^Department of Oral Surgery, Dental School, Araraquara University (UNIARA), Araraquara 14801-340, Brazil; ^3^Department of Orthodontics, School of Dentistry, Sao Leopoldo Mandic, Campinas, São Paulo, Brazil; ^4^Department of Social Dentistry, School of Dentistry, São Paulo State University (UNESP), Araraquara, São Paulo 14801-930, Brazil; ^5^Department of Dental Materials and Prosthodontics, School of Dentistry, São Paulo State University (UNESP), Aracatuba, São Paulo 16015-050, Brazil

**Keywords:** bone graft, dental agenesis, dental implant, oral rehabilitation, orthodontics

## Abstract

Congenital dental agenesis, particularly in younger adults, can have a profound impact on aesthetics and overall quality of life. The scientific literature offers various management strategies for this condition, with orthodontic movement and implant-supported rehabilitation being central to treatment. However, achieving predictable and successful outcomes necessitates a comprehensive multidisciplinary approach. Such an approach integrates diverse professional perspectives to ensure accurate diagnosis, prognosis, and treatment planning, ultimately aiming to restore dental function and address aesthetic concerns effectively. In this case report, we present the successful rehabilitation of a young patient with congenital agenesis of the mandibular central incisors. The treatment strategy combined oral surgery (extraction of deciduous teeth and autogenous bone graft), orthodontic movement (opening spaces to allow implant installation), periodontics (connective tissue graft), implantology, and prosthetic planning. We detail the specific surgical approaches employed and discuss how their integration contributed to the overall success of the case. This multidisciplinary treatment approach not only restored dental function but also met the patient's aesthetic expectations and enhanced the patient's quality of life, highlighting the importance of a coordinated approach in managing complex dental conditions.

## 1. Introduction

Dental agenesis stands out as the most prevalent developmental anomaly in dentistry [1, 2], yet its underlying etiology remains incompletely understood. Evidence suggests a combination of environmental and genetic factors at play, with potential interactions between them. According to Wang et al. [3], the bulk of dental agenesis cases can be linked to genetic factors and DNA methylation. Zhou et al. [4] showed that chemotherapy, radiotherapy, metabolic imbalances, infections, and diseases might affect anterior deciduous teeth potentially contributing to this anomaly.

Studies indicate that dental agenesis affects approximately 20% of the world's population [5], showing a higher prevalence among females [6–8]. Furthermore, its occurrence may vary based on geographic location [7–9]. The most commonly affected teeth, excluding third molars, are the lower second premolars, followed by the upper lateral incisors, upper second premolars, and lower incisors [6, 10, 11]. This condition can impact significantly the facial esthetics, mainly influenced by teeth and eyes, potentially affecting psychological well-being, particularly during adolescence [10]. Dental agenesis also compromises aesthetic, occlusal, and masticatory functions [5, 12, 13].

Early diagnosis and effective management are essential for preventing complications like malocclusion and local bone atrophy [13, 14]. When dental agenesis is detected in the anterior region, a thorough assessment considering the patient's age, facial profile, type of malocclusion, and tooth alignment is crucial for developing a comprehensive treatment plan [10].

Numerous therapeutic options exist for rehabilitation of dental agenesis [6], including orthodontic treatments for space closure or opening [10], as well as the installation of removable, fixed, or implant-supported prostheses [15]. However, the literature lacks consensus on the ideal treatment for these cases [16], demanding a multidisciplinary team approach to select the most suitable option based on their expertise and the patient's expectations, to achieve long-term aesthetic and functional outcomes effectively [17].

Space closure is increasingly recognized as a promising treatment strategy for adolescent patients, aiming to complete orthodontic treatment more swiftly. This approach facilitates the patient's ability to adjust to and sustain the final occlusion while their craniofacial development is ongoing [16–18]. The primary advantage of this method lies in its potential to accelerate the overall treatment process, allowing for earlier achievement of the desired occlusal outcome. However, the decision to pursue space closure must be based on a comprehensive case evaluation. A thorough assessment is essential to determine the suitability of this treatment option for each individual patient. Factors such as the patient's current craniofacial growth stage, the specifics of the dental issue at hand, and the long-term implications for occlusion and overall oral health need to be carefully considered. Only through a detailed and individualized evaluation can the feasibility of space closure be accurately determined and optimized for the best possible outcome.

Nowadays, fixed prosthesis is not frequently indicated due to the necessity of adjacent teeth preparation for prosthesis installation [16]. The most reliable and indicated treatment strategy is the placement of dental implants, as a more conservative approach, by preserving the adjacent teeth and the alveolar bone crest height and width [19]. However, a thorough case evaluation is crucial to determine the feasibility of this treatment option, considering factors like available space, need for prior orthodontic treatment, and volume of bone and soft tissue, all of which influence the final aesthetic result [16]. In cases of bone atrophy, bone grafting is recommended to facilitate the ideal three-dimensional positioning of the implant [15]. Bone graft options vary among autogenous, homologous, or xenogenous grafts based on the patient's specific situation and demands [15, 20, 21].

Scientific advancements have significantly improved surgical and prosthetic treatments, enhancing long-term stability, optimizing aesthetic and functional outcomes, and reducing treatment time for patients [21]. Congenital absence of the mandibular incisor is not a common condition posing a challenge, particularly in younger patients due to high aesthetic demands [11, 18]. Therefore, this article presents a case of mandibular central incisors replacement due congenital agenesis of permanent teeth in a young patient. It underscores the importance of a comprehensive and personalized multidisciplinary approach, which effectively resolved the case and will be further discussed below.

## 2. Case Presentation

### 2.1. Etiology and Diagnosis

**Figure 1:**
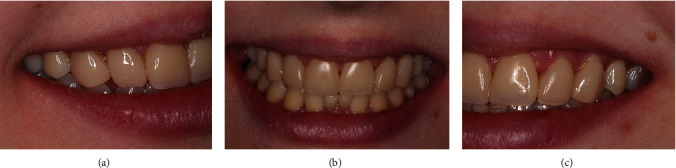
(a) Intraoral photograph and smile esthetics showcasing periodontal health. (b, c) Lateral views of the smile.

**Figure 2:**
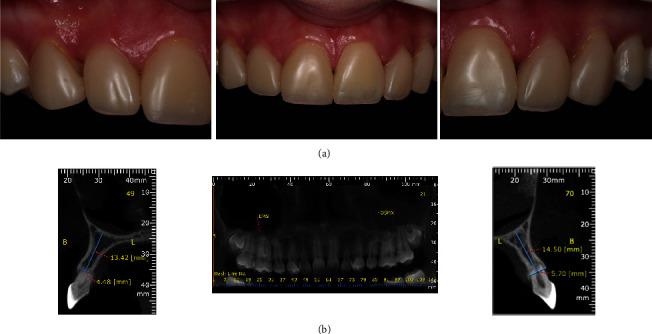
(a) Intraoral images of the anterior teeth. (b) Radiographic examination revealing the absence of canines, extensive root resorption of the upper lateral incisors, incomplete odontogenesis of the impacted third molars, and bilateral congenital agenesis of the lower incisors.

**Figure 3:**
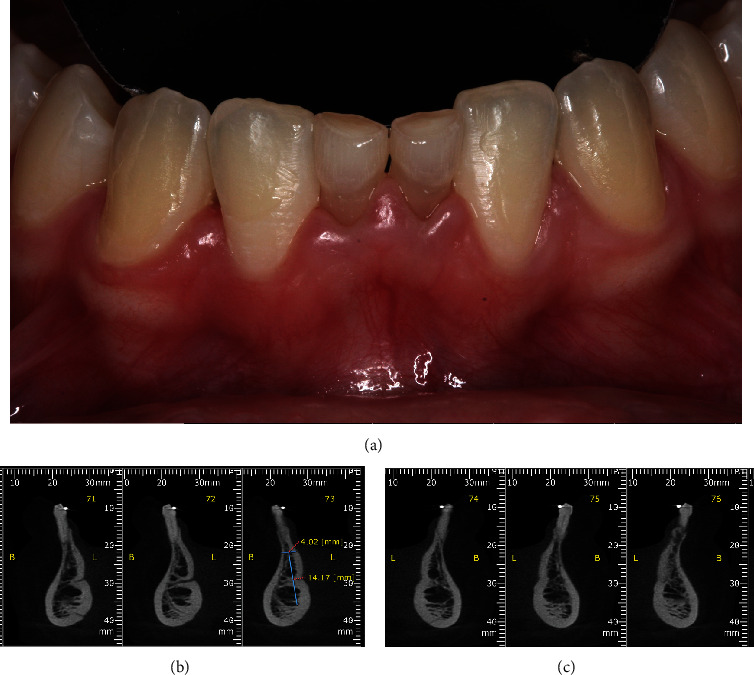
(a) Clinical image of the lower anterior region showing the presence of deciduous central incisors in the mandible with limited space for replacement of both teeth with dental implants. CBCT scan revealed a bone height of 14.17 mm and width of 4.02 mm in the anterior mandibular region; (b) a bone height of 13.42 mm; (c) width of 4.48 mm in the right upper lateral incisor; and a bone height of 14.50 mm and width of 5.70 mm in the left upper lateral incisor.

A 17-year-old female patient sought dental treatment to improve her smile's aesthetics. In her medical history, the patient reported undergoing surgery approximately 4 years ago to remove impacted upper canines and to mesially move her first premolars. Furthermore, she denied using alcohol and tobacco, and her systemic condition was unremarkable. Intraoral and radiographic (Figures 1, 2, and 3) examinations revealed the presence of deciduous lower central incisors, upper lateral incisors with 2/3 root resorption, and old resin restorations on some anterior teeth in the upper region, negatively affecting the smile's aesthetics, which was the patient's primary concern.

However, the patient demonstrated a high standard of plaque control, indicating satisfactory gingival and periodontal health (Figure 1). Based on the clinical and radiographic examination, our team proposed replacing the deciduous teeth with dental implants to the patient. However, in order to be able to place the implants in the correct location, orthodontic treatment was also indicated to open the necessary space allowing the placement of a correct implant diameter. The treatment plan was presented to the patient, who authorized its execution.

Cone beam computed tomography (CBCT) was performed to assess the alveolar bone characteristics (available bone thickness and height) for implant placement (Figures 2 and 3). Tomographic data obtained in the lower region showed a bone height of 14.17 mm and a width of 4.02 mm. In the upper region (right lateral incisor), 13.42 mm of bone height and 4.48 mm of width were observed; and in the left lateral incisor, with a height of 14.50 mm and a width of 5.70 mm. The treatment plan for this case comprises several steps: extraction of the upper lateral incision and lower central incisors, bone grafting augmentation procedures, orthodontic movement, dental implant placement, and finally, case rehabilitation with a fixed-implant prosthesis.

### 2.2. Treatment Objective

The main treatment goals were to correct the bite, establish stable Class I canine and Class II molar occlusal relationships, placement of dental implants in the correct tridimensional position, enhance the soft tissue phenotype and the width of the alveolar crest, and improve the smile's aesthetics trough oral rehabilitation with definitive implant-supportive prosthesis.

### 2.3. Treatment Progress

**Figure 4:**
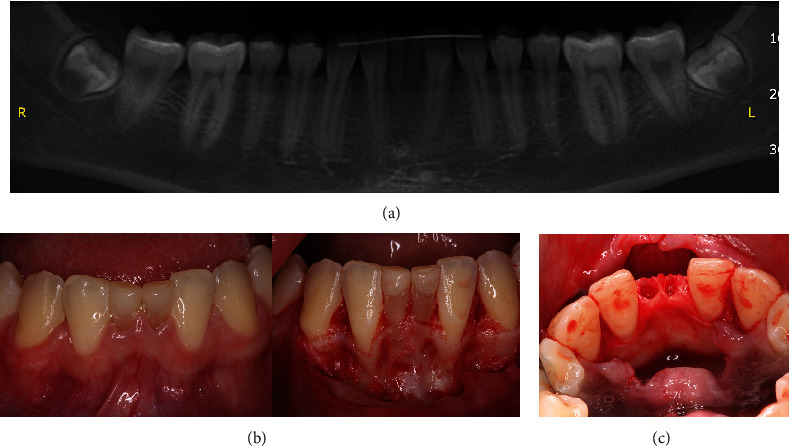
(a) Panoramic image from the lower arch. (b) Mucoperiosteal flap exposing the mandibular deciduous teeth. (c) Postsurgical aspect.

**Figure 5:**
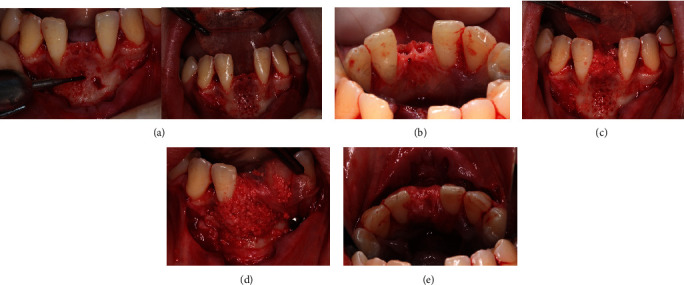
(a) Decortication of the mandibular vestibular wall using a cylindrical bur to enhance blood supply. (b) Following the extraction of the third molar, bone tissue was removed from the mandibular ramus with an osteo-collecting bur. (c–e) Autogenous bone graft inserted and collagen membrane positioned.

**Figure 6:**
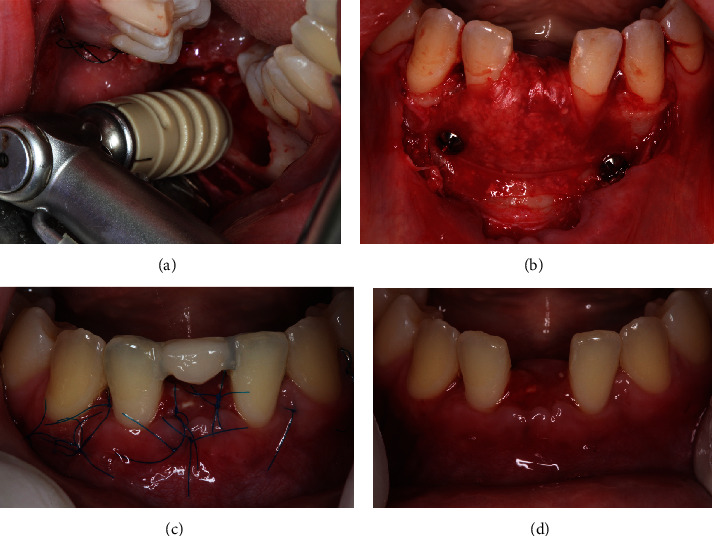
(a) Trephine bur to harvest the autogenous bone from the third molar area; (b) fixation of the collagen membrane to aid stability during insertion with a miniscrew; (c) suture with nylon 6-0 thread, resulting in minimal tissue reaction; (d) tissue aspect 2 weeks post healing in the recipient area.

Initially, the mandibular anterior region was anesthetized with infiltrative anesthesia (lidocaine 2% and epinephrine 1:100,000). Then, a sulcular incision was made, extending from the left to the right canine, followed by the elevation of a mucoperiosteal flap to expose the entire alveolar bone crest, facilitating the extraction of the deciduous teeth (Figure 4). After the extraction, it became evident that the vestibular bone plate of the region was thin, hindering the proper three-dimensional orientation for implant placement. To address this issue, bone decortication was performed using a cylindrical drill to stimulate cell recruitment to the region and facilitate bone grafting. Immediately after decorticalization, autogenous bone grafting was harvested from the mandibular ramus (after extraction of the mandibular third molar) using a specific trephine drill (Figures 5 and 6).

Subsequently, the harvested autogenous bone graft was gently placed over the central incisor region and covered with a collagen membrane (Bio-Gide, Geistlich, Switzerland) to maintain the particulate bone in place and to prevent the migration of epithelial cells during the healing process. Finally, the membrane was fixed with two screws, and the flap was repositioned and sutured with interrupted stitches using 6-0 nylon thread (Techsuture, Bauru, São Paulo, Brazil) (Figure 6). Two weeks after surgery, the sutures were removed and the healing was uneventful (Figure 6).

**Figure 7:**
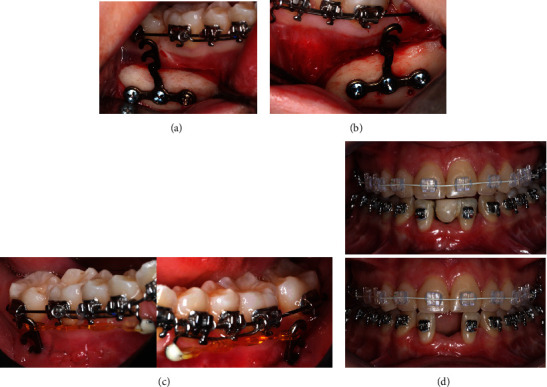
(a, b) Miniplates installed with the mucoperiosteal flap, exposing only the area of their insertion on the mandibular body; (c) appearance of the miniplates installed and tissue healing in the flap region; and (d) temporary crown sectioned on the right side to allow for dental movement of the incisors.

A 4-month interval preceded the start of orthodontic treatment, during which the patient maintained provisional restorations connected to the adjacent teeth. The orthodontic phase involved the placement of T miniplate in the posterior mandibular region to assist in the distalization of the canines, ensuring stable and efficient movement (Figure 7).

**Figure 8:**
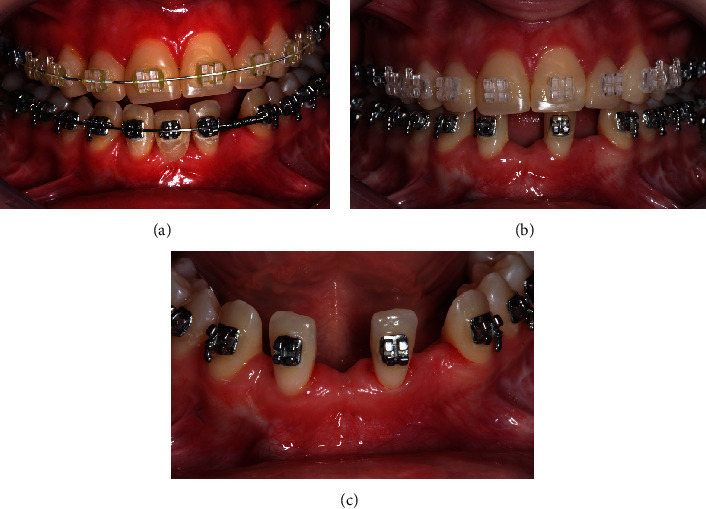
(a) Mesialization of the left incisor to allow for implant placement between the left incisor and canine; (b) the dental movement enabled parallelism of the incisor; and (c) visualization of the planned space for dental implant installation.

Once a Class I canine relationship was achieved after distal movement, the right lateral incisor was moved distally using a NiTi open spring to optimize crown/root positioning, aligning it parallel to the left lateral incisor, which was moved mesially to occupy the central incisor area, creating space in the lateral incisor region for implant placement (Figure 8). After the completion of orthodontic treatment, there was sufficient space (mesiodistal) to install two implants, one in the right central incisor region and another in the left lateral incisor region.

**Figure 9:**
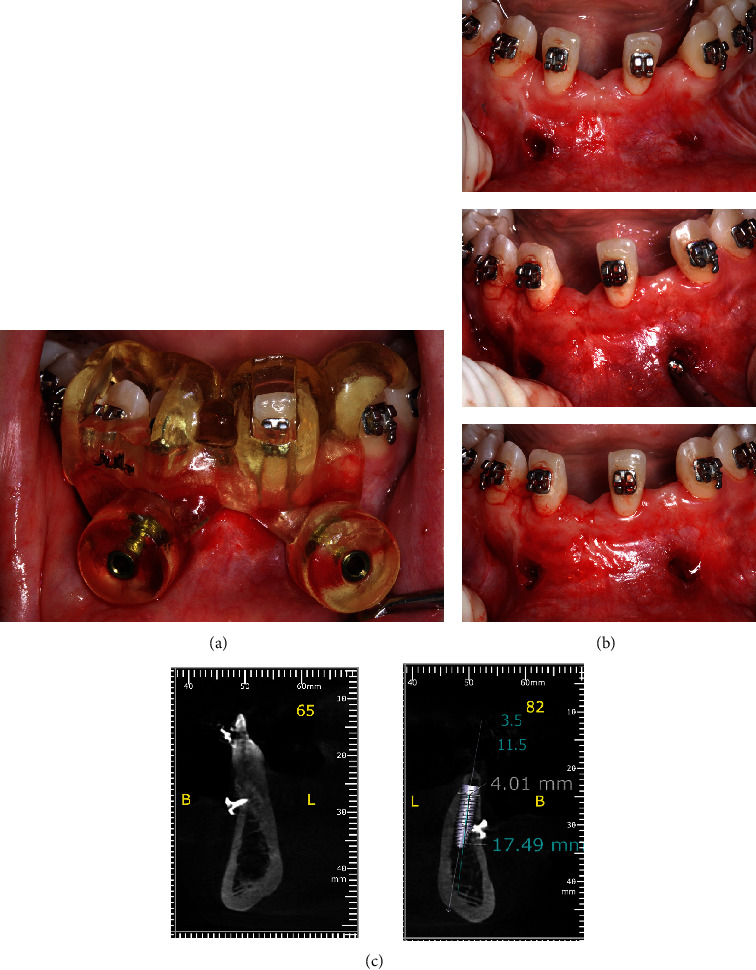
(a) Surgical guide positioned for implant placement; (b) removal of a miniscrew prior to implant placement for subsequent imaging examination; and (c) CBCT imaging revealing the width of the alveolar crest allowing the placement of a proper diameter implant.

**Figure 10:**
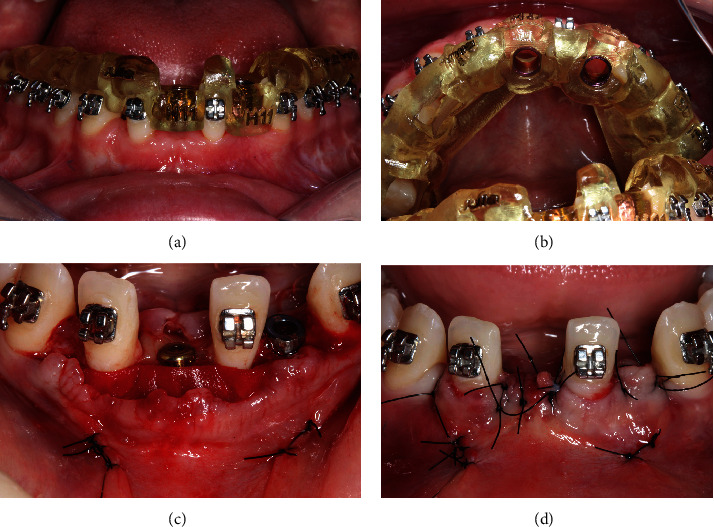
(a–c) Placement of two dental implants to replace the missing teeth. (d) Suture with 5-0 nylon thread, using interrupted stitches.

A CBCT scan also confirmed an increase in the thickness of the buccal bone plate after the grafting, enabling correct implant placement. Subsequently, the two screws that secured the membrane were removed, and with the aid of a surgical guide, two dental implants (Neodente, Curitiba, PR, Brazil; 3.5 × 11.5 mm) were installed after raising the mucoperiosteal flap, followed by the placement of cover screws on the implants. Finally, the flap was repositioned to its original location and sutured with interrupted stitches using 5-0 nylon thread (Techsuture, Bauru, São Paulo, Brazil) (Figures 9 and 10).

**Figure 11:**
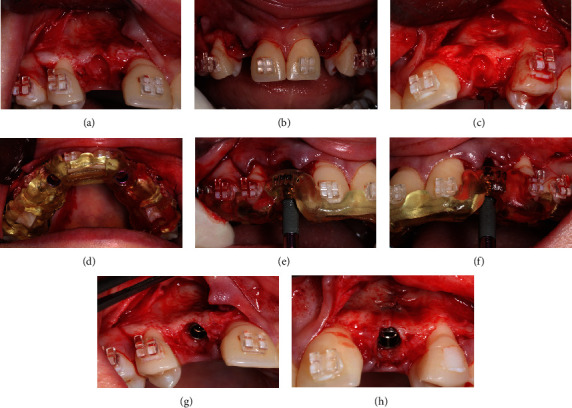
(a) Surgical procedures for implant placement in the maxillary arch. Mucoperiosteal flap was raised to expose the alveolar bone crest; (b, c) aspect of the alveolus after extraction of the right and left lateral incisor; (d) surgical guide positioned; (e–h) right and left implant installation with the assistance of the surgical guide for proper positioning.

For the upper region management, the patient underwent extraction of the lateral incisors after the elevation of the mucoperiosteal flap (Figure 11). With the aid of a surgical guide, two implants (Neodente, Curitiba, PR, Brazil; 4.0 × 11.5 mm) were installed (Figures 8(d), 8(e), and 8(f)), maintaining the necessary distance between the adjacent teeth to ensure complete filling of the papilla in the interproximal region (Figure 11).

**Figure 12:**
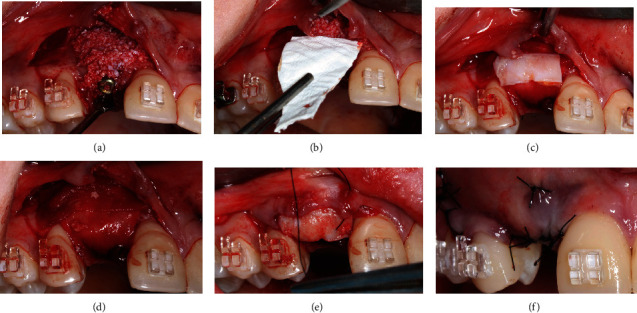
(a) Xenogenous bone graft (bovine bone mineral—BioOss) positioned on the buccal wall; (b, c) followed by the placement of a collagen membrane; (d) insertion of autogenous connective tissue graft removed from the palatal area; (e) sutures with interrupted stiches using 5-0 nylon thread; and (f) immediate postoperative period.

**Figure 13:**
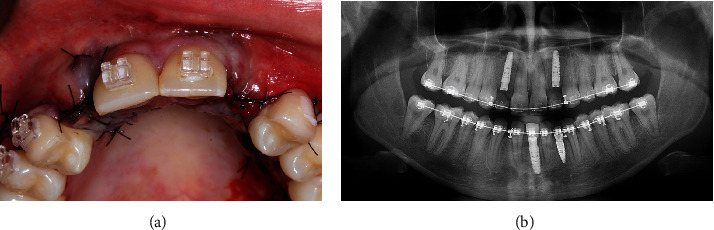
(a) Immediate postoperative view. (b) Radiographic examination showing the parallelism and osseointegration of the dental implants and bone healing.

After implant placement, a bovine bone substitute graft (BioOss, Geistlich, Switzerland) was applied to the vestibular bone on the right side to enhance bone width, followed by the placement of a collagen membrane over the graft. To increase the thickness of the keratinized gingiva, a connective tissue graft was harvested from the palatal area and positioned over the collagen membrane (Bio-Gide, Geistlich, Switzerland) (Figure 12). Subsequently, the soft tissue graft was sutured in place, and the mucoperiosteal flap was repositioned and sutured with 5-0 nylon sutures (Techsuture, Bauru, São Paulo, Brazil) (Figure 13).

The dental implants in the upper and lower arches were left unloaded to facilitate implant osseointegration for 6 months. During this entire healing period, the patient received temporary crowns fixed to the orthodontic arch, resulting in satisfactory aesthetic results.

### 2.4. Treatment Outcomes

**Figure 14:**
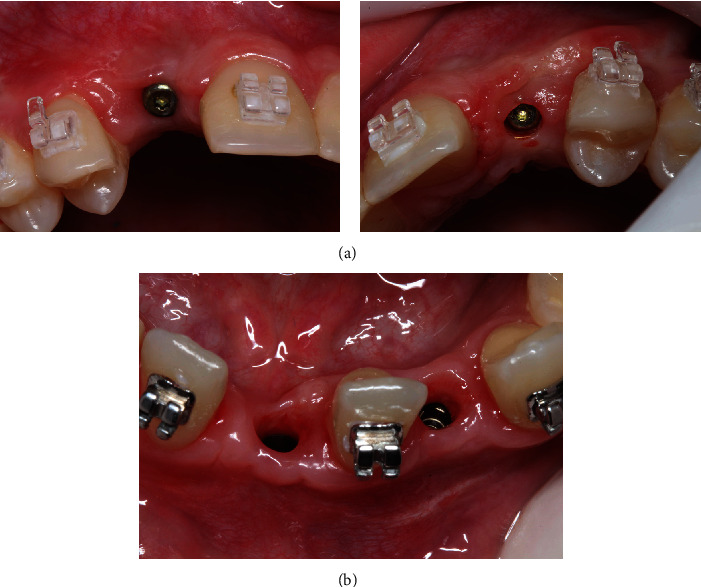
(a) Occlusal view of the lower arch showing the healing process and (b) the periapical radiograph demonstrating the correct placement of the implants.

**Figure 15:**
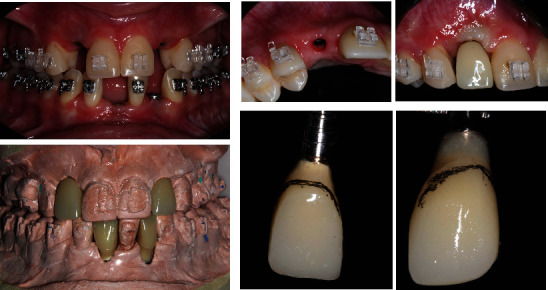
Clinical view of the maxillary and mandibular arches demonstrating healthy gingival tissue, with thick gingival phenotype around the implant.

**Figure 16:**
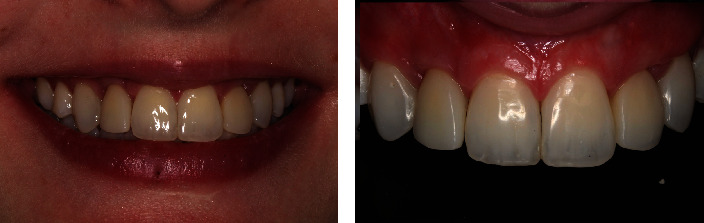
Prosthetic procedures to fabricate the porcelain crowns.

The patient's surgical healing progressed without complications. Radiographic examination reveal the correct placement of the implants in both arches, respecting the minimal distances to the adjacent tooth (2 mm) (Figures 13 and 14). After a 6-month period, the upper and lower implants were reopened to expose the cover screws with minimally invasive surgery (Figure 14), and prosthetic procedures were initiated (Figure 15). Prior to fabricating the definitive crowns, dental bleaching was performed on both arches using a combination technique. Following the successful bleaching outcome, metal-free porcelain crowns were fabricated and installed (Figure 16).

**Figure 17:**
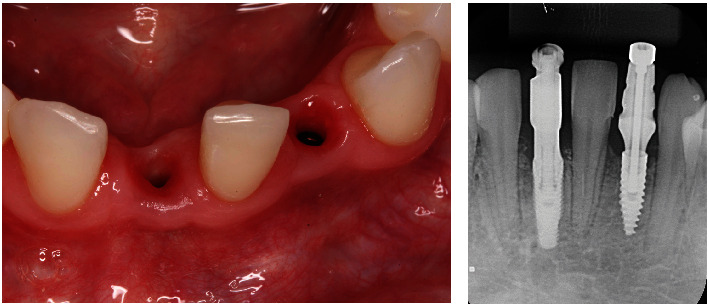
Clinical view of the smile in the upper region after 2 years posttreatment showing excellent esthetic results.

**Figure 18:**
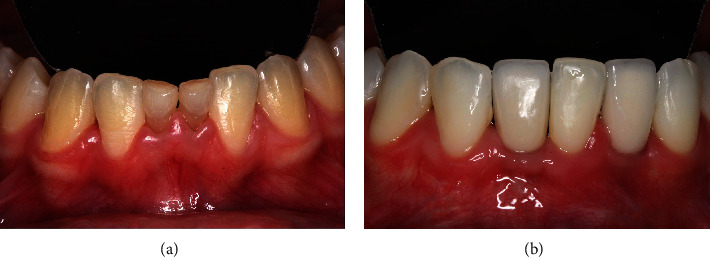
(a) Before and (b) after treatment in the lower region with the substitution of the deciduous teeth by dental implants after orthodontic movement.

**Figure 19:**
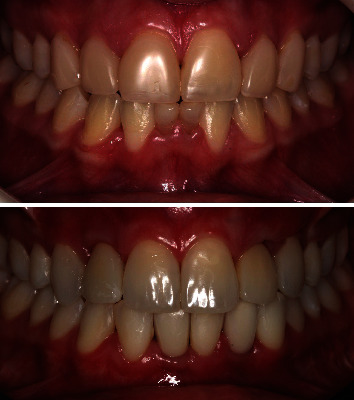
Final appearance of the smile after teeth whitening, installation of the definitive crowns, and fabrication of aesthetic restorations.

A two-year follow-up showed a stable occlusion characterized by evenly distributed occlusal contacts. Furthermore, there was bone gain, with the implants positioned ideally in the three-dimensional positioning. The smile appeared harmonious, with a reduced buccal corridor, resulting in a broader smile, and the patient expressed satisfaction with the outcome (Figures 17, 18 and 19).

## 3. Discussion

Several therapeutic alternatives are available for the complete restoration of the smile in cases of agenesis. Simplistically, these options include closure, maintenance, or redistribution of the available local space [22, 23]. In this case, redistribution of the available space followed by rehabilitation with dental implants was chosen to achieve long-term aesthetic and functional excellence [1, 24]. Adjunctive treatments, that is, bone augmentation procedures, autogenous connective tissue graft, and the placement of a collagen membrane were carefully considered, demonstrating a comprehensive approach and a detailed multidisciplinary treatment plan [16, 25].

To allow the proper installation of implants with convenient diameter and length, careful preparation of the remaining space is fundamental. Therefore, orthodontic treatment was recommended as a crucial step to create and redistribute the necessary spaces, ensuring positioning, stability, and aesthetics of the planned implants [1, 16, 25]. Another important factor is the width of the alveolar crest [25]. Hence, autogenous bone grafting in the anterior mandibular region was chosen for two primary reasons. First, it addresses biological limitations, as dental implants require 1.5–2 mm of buccal bone around them [25, 26] and 3–4 mm in the mesiodistal direction to ensure adequate blood supply and reduce the risk of bone resorption [26]. Second, it minimizes the risk of root resorption. Studies such as those by Dueled et al. [24] demonstrate that orthodontic movement in cases of dental agenesis increases the risk of root resorption, as dental movement can reach the cortical bone wall due to the lower amount of trabecular bone. The reconstruction of the atrophic alveolus with bone grafting prior to implant placement, as described by Lu et al. [27] is fundamental for safe and effective orthodontic movement [24]. Among several bone grafting alternatives, autogenous bone grafting was indicated in this case, taking advantage of the same surgical procedure as the extraction of the third molar. This approach offers some advantages, including reduced surgical time, decreased patient morbidity, and the utilization of readily available bone tissue [1, 28].

It is worth noting that for orthodontic planning in these cases, other factors besides the atrophy of the alveolar process should be considered. These include the available space for rehabilitation, the position of the incisors in the dental arch, the type of malocclusion according to Angle's classification, dental symmetry, and the individual's facial profile [6, 18]. In this case, the patient exhibits a flat facial profile with Class III malocclusion due to mesialization of the teeth adjacent to the agenesis. The bulky canines and narrow buccal corridor indicated that dental movement to open space was the best treatment option for rehabilitation [6].

To ensure adequate space for future implants, T-type miniplates were chosen as the ideal option. Fixation was achieved using three screws, providing increased stability during traction movement, reducing the risk of failure, and minimizing bone stress through optimized force distribution. This approach ensured smooth movement and reduced stress on the alveolar bone [29]. Miniplates are orthodontic anchorage devices that allow tooth movement without damaging adjacent structures, such as the roots of other teeth. Their compatibility with fixed orthodontic appliances ensures greater control and precision in tooth movements, while their optimized design provides maximum comfort for the patient during treatment. It is important to note that, like any device installed in the oral cavity, miniplates require both pre- and postoperative care, including strict patient hygiene control to prevent the accumulation of bacterial plaque and possible infections [30–32]. After achieving the desired space through fixed orthodontics, the implant installation stage was performed. The fabrication of a surgical guide was essential to ensure the ideal three-dimensional positioning of the implants, providing maximum predictability for the case [27].

In the maxilla, immediate implant placement using guided surgery was chosen to replace the lateral incisors. Due to advanced resorption of the alveolar crest, a mucoperiosteal flap was raised exposing the entire bone area, followed by the immediate placement of the implants. Then, reconstruction of the bone walls was performed using xenogenous bone graft to increase the bone width around the implant, followed by the placement of a collagen membrane [33–35]. This approach stimulates bone growth in the implant region, promoting optimal support for osseointegration and long-term implant stability. Studies indicate that using bovine bone particle grafts in conjunction with a collagen membrane positively contributes to bone regeneration at the defect site [36–39].

To ensure optimal aesthetics and functionality, the thickness of the peri-implant soft tissue should not be less than 2 mm [40, 41]. In this case, a thin peri-implant mucosa was diagnosed. To increase the peri-implant phenotype, connective tissue graft was harvested from the palatal area. Connective tissue grafting is highlighted in the literature for its importance in maintaining peri-implant tissue health and marginal bone stability around dental implants [40, 41]. The connective tissue graft increases the thickness of the peri-implant mucosa, prevent gingival recession, facilitate the oral hygiene practices, and ensure high aesthetic outcomes [42–44]. The thickness gain obtained with the connective tissue graft can vary between 0.71 and 2.08 mm [40, 41]; the greater the amount of mucosa, the better the emergence profile of the restoration, contributing positively to a stable aesthetic-functional outcome. Tissue integration results are typically achieved after 3 months of postoperative care [44].

Beyond local factors, the patient's chief complaint is a fundamental element in treatment planning [8]. The ultimate goals in cases of dental agenesis are twofold: to restore stable occlusion and to contribute to a harmonious smile aesthetic. Therefore, it is essential that all stages, from planning to execution, consider smile aesthetics to achieve a natural and pleasing result for the patient [1, 23]. In modern dentistry, success hinges on individualized planning that takes into account specific local factors, as well as the patient's main concerns and effective communication among professionals from various specialties. This approach ensures that the treatment plan is tailored to meet the individual's expectations and requirements. With the collaboration of a multidisciplinary team, dental agenesis can be effectively addressed, leading to the restoration of smiles, boosting self-esteem, and enhancing the patient's quality of life.

## 4. Conclusion

This case report underscores the significance of a comprehensive multidisciplinary approach in managing congenital dental agenesis, particularly in younger patients. The successful rehabilitation of a 17-year-old female with agenesis of the mandibular central incisors highlights the effective integration of oral surgery, orthodontics, periodontics, implantology, and prosthetic planning. The treatment strategy involved a series of well-coordinated steps: extraction of deciduous teeth, autogenous bone grafting, orthodontic space creation, and precise implant placement followed by prosthetic restoration. This approach not only addressed the patient's aesthetic concerns but also restored dental function, demonstrating the critical role of personalized, team-based care in achieving optimal outcomes. The use of autogenous bone grafting and connective tissue grafting, alongside advanced orthodontic techniques and guided implant surgery, contributed to the successful enhancement of both functional and aesthetic aspects of the patient's smile. The positive outcomes observed in this case, including stable occlusion, harmonious smile aesthetics, and patient satisfaction, affirm the efficacy of a multidisciplinary treatment plan in managing complex dental conditions.

## Data Availability

The data that support the findings of this study are available from the corresponding author upon reasonable request.
